# Editorial Notes

**Published:** 1932

**Authors:** 


### Editorial Notes

**Death of Dr. Carey Coombs.**

The sudden death of Dr. Carey Coombs has deprived the *Journal* of its Assistant Editor. His services in this capacity have been invaluable. He had had long experience as Editor of the *Medical Annual* before he joined the Editorial Committee of this *Journal* in 1919; he became Assistant Editor in 1926. His diligence and judgment in obtaining original articles for the *Journal* added greatly to its interest, and his wide knowledge of medical journalism in general was always at its service. He was a genial and helpful colleague, whose loss is very widely deplored, and by none more than his fellow-workers on the Editorial Committee of this *Journal.*

**Medical Dinner.**

The Annual Medical Dinner was held on Thursday, 8th December, at the Clifton Zoological Gardens, Mr. C. Ferrier Walters being in the Chair. The guest of the evening was Dr. J. R. Charles, Consulting Physician to the Bristol Royal Infirmary. The attendance was unusually large and included a great number of Dr. Charles's former clinical clerks, whose enthusiasm gave evidence of their cordial regard for him. The Dean (Professor Fawcett) gave notice of some arrangements for the Centenary Celebrations, the details of which follow.

**Centenary of the Medical School.** THE Medical School of the University of Bristol will celebrate its Centenary on 30th June and 1st July, 1933. A provisional programme has been arranged which will include, on the afternoon of the first day, receptions at the Bristol Royal Infirmary and Bristol General Hospital, in the evening a dinner of present and past students and guests, to be followed by a reception at the University, and on the second day a combined graduation ceremony of ordinary and honorary graduands, followed by a luncheon. A more detailed statement will appear later.

